# Anemia during pregnancy and adverse pregnancy outcomes: a systematic review and meta-analysis of cohort studies

**DOI:** 10.3389/fgwh.2025.1502585

**Published:** 2025-01-31

**Authors:** Rui Wang, Shan Xu, Xiaoyu Hao, Xingyi Jin, Da Pan, Hui Xia, Wang Liao, Ligang Yang, Shaokang Wang

**Affiliations:** ^1^ Key Laboratory of Environmental Medicine and Engineering of Ministry of Education, and Department of Nutrition and Food Hygiene, School of Public Health, Southeast University, Nanjing, China; ^2^ Clinical Medical Research Center for Plateau Gastroenterological Disease of Xizang Autonomous Region, and School of Medicine, Xizang Minzu University, Xianyang, China

**Keywords:** anemia, anemia in pregnancy, pregnancy outcome, cohort study, maternal health

## Abstract

**Objectives:**

Anemia in pregnancy has been a topic of interest for researchers due to its potential impact on various adverse pregnancy outcomes. This study aims to explore the relationship between anemia and adverse pregnancy outcomes such as preterm birth, low birth weight, and maternal mortality.

**Methods:**

We conducted both a systematic review and a meta-analysis on the associations between anemia during pregnancy and adverse pregnancy outcomes. We searched Chinese databases (CNKI, Wanfang, CBM, VIP) and English ones (Cochrane Library, PubMed, Embase, Web of Science). Two researcher-authors independently assessed study quality with the Newcastle-Ottawa Scale. After extracting data, we analyzed heterogeneity and used a random-effects model for higher heterogeneity and a fixed-effects model for low heterogeneity in the meta-analysis while also systematically synthesizing and narratively describing findings in the systematic review.

**Results:**

A total of 31 cohort studies were included. Meta-analysis showed that the risk of postpartum hemorrhage [RR [95% CI], 2.76 [1.63, 4.66]], premature rupture of membranes (PROM) [1.94 (1.26, 3.00)], preterm delivery [1.51 (1.33, 1.72)], low birth weight (LBW) [1.40 (1.19, 1.63)], cesarean section[1.33 (1.02, 1.74)], gestational hypertension[1.28 (1.14, 1.44)] and neonatal asphyxia[1.21 (1.07, 1.37)] was higher in the group of anemia in pregnancy than in the control group.

**Conclusion:**

Maternal anemia is associated with an increased risk of seven adverse pregnancy outcomes: postpartum hemorrhage, PROM, preterm delivery, LBW, cesarean section, gestational hypertension and neonatal asphyxia. Appropriate nutritional supplementation and screening for anemia before and during pregnancy are recommended to improve maternal health and manage adverse pregnancy outcomes.

## 1 Introduction

Anemia during pregnancy is a significant global health concern that affects a substantial number of women worldwide, with a prevalence rate of 30% among women of childbearing age (1). It is defined as a condition where hemoglobin levels are below average, resulting in a decreased oxygen-carrying capacity of red blood cells in tissues (2). During pregnancy, as the fetus grows, pregnant women experience metabolic changes, hormonal level fluctuations and an increase in blood volume, and the physiological demands for iron, folate, and other nutrients increase significantly to support the growth and development of the fetus, as well as to meet the expanded blood volume requirements of the mother (3–6). This heightened need often places pregnant women at a higher risk of developing anemia if these nutrients are inadequately supplied through diet or if there are underlying health conditions that interfere with nutrient absorption or utilization (7, 8). In developing countries, where access to proper nutrition and prenatal care may be limited, the rates tend to be even higher (9). This not only impacts the health and well-being of the mother but also has far-reaching implications for the pregnancy itself and the health of the newborn. Severe anemia during pregnancy can have both short-term and long-term effects on the mother, fetus and newborn, such as leading to the occurrence of adverse pregnancy outcomes like gestational hypertension, miscarriage, preterm birth and low birth weight infants (10, 11).

Despite the well-documented associations between anemia during pregnancy and these adverse outcomes, there remains some variability in the reported findings across different studies. This could be attributed to differences in the diagnostic criteria used for anemia, variations in study populations, and disparities in the methods employed to measure and assess adverse pregnancy outcomes. Furthermore, the complex interplay of multiple factors such as socioeconomic status, access to healthcare, and coexisting medical conditions makes it challenging to fully understand the precise mechanisms through which anemia leads to these adverse events. Given these uncertainties and the significant impact on both maternal and neonatal health, this study conducts a systematic review and meta-analysis of prospective and retrospective cohort studies published domestically and internationally to explore the relationship and impact degree between anemia during pregnancy and various adverse pregnancy outcomes.

## 2 Methods

### 2.1 Literature search

Computer searches were conducted in Chinese databases and English databases: CNKI (China National Knowledge Infrastructure), Wanfang, VIP (Chongqing VIP Information Co., Ltd.), CBM (China Biology Medicine disc), Cochrane Library, PubMed, Embase and Web of Science. A search strategy combining both MeSH terms and free-text terms was used. The complete search strings used in English databases is (“Anemia” OR “Anaemia” OR “Iron Deficiency Anemia”) AND (“Pregnancy” OR “Pregnant Women”) AND (“PROM” OR “SGA” OR “Pre-eclampsia” OR “Fetal Distress” OR “Preterm Birth” OR “Premature Delivery” OR “Neonatal Asphyxia” OR “Low Birth Weight” OR “Postpartum Hemorrhage” OR “Cesarean Section” OR “Gestational Hypertension” OR “Adverse Pregnancy Outcomes”). The search strategy used in different databases is different, but the basic keywords and search logic are the same.

### 2.2 Inclusion criteria

(1) Cohort study literature on anemia and pregnancy outcomes published from the establishment of the database until December 2023; (2) The researchers excluded multiple pregnancies from the sample at the time of design; (3) Studies must include outcomes for pregnant mothers in both the anemia group and the control group, as well as birth outcomes for newborns; (4) Outcome variables include gestational hypertension, gestational diabetes mellitus (GDM), puerperal infection, cesarean section, premature rupture of membranes (PROM), preterm birth, postpartum hemorrhage, congenital malformations, fetal distress, neonatal asphyxia, low birth weight (LBW).

### 2.3 Exclusion criteria

(1) Research on specific types of anemia, such as thalassemia or aplastic anemia; (2) Studies without a control group or with weak comparability (studies not adhering to the diagnostic criteria or using other standards for diagnosis); (3) Duplicate reports or studies of poor quality.

### 2.4 Diagnostic criteria

According to the World Health Organization (WHO) “Hemoglobin Concentration for the Diagnosis and Severity Assessment of anemia” released in 2011, pregnant women with hemoglobin concentrations less than 11 g/dl can be diagnosed as anemia (12). The Indian Council of Medical Research (ICMR) established the following criteria in 1989: Very severe anemia: hemoglobin level < 4 g/dl; Severe anemia: 4 g/dl ≤ hemoglobin level < 6.9 g/dl; Moderate anemia: 7 g/dl ≤ hemoglobin level < 9.9 g/dl; Mild anemia: 10 g/dl ≤ hemoglobin level < 10.9 g/dl (13). The diagnostic criteria for anemia are based on either WHO standards or ICMR standards, depending on the content of the studies.

### 2.5 Literature screening and data extraction

To avoid bias in literature screening, two researchers involved in the study independently conducted a preliminary screening of the literature by reading abstracts based on the search strategy and inclusion/exclusion criteria. Anemia in pregnancy is not a rare disease, and when the sample size is small, it does not achieve the ideal statistical test efficacy (14). For these reasons, we excluded studies with small sample sizes (*n* < 85), incomplete information or data, unclear statistical methods, or duplicate reports due to poor quality. After completing the screening independently, they compared their selections. For any literature with disagreement on inclusion, consensus was reached through discussion.

### 2.6 Quality assessment

Two evaluators with relevant expertise independently assessed the quality of the included studies using the Newcastle-Ottawa Scale (NOS) for assessing the quality of non-randomized studies (15). For any literature where there was disagreement in the assessment, consensus was reached through discussion. The NOS scale has a total score of 9 points, with studies scoring seven or above considered higher quality and eligible for inclusion in the systematic review and meta-analysis.

### 2.7 Statistical analysis

Meta-analysis was conducted using RevMan 5.0. The first step involved testing for heterogeneity among the included studies, which refers to the consistency or trend of the results across the studies, indicated by the *I^2^* value. The *I^2^* values are 25%, 50%, and 75%, respectively, representing low, medium, and high degrees of heterogeneity. The relative risk (*RR*) was used to compare the combined effect. If *I^2^* < 50%, a fixed-effect model was employed for analysis; otherwise, a random-effect model was used. Meta-regression was utilized for sensitivity analysis and subgroup analysis to identify potential sources of heterogeneity. Forest plots were used to display the *RR* values and 95% *CI* of factors from the included studies, and funnel plots were employed to understand potential publication bias.

## 3 Results

### 3.1 Literature search and study selection

**Figure 1:**
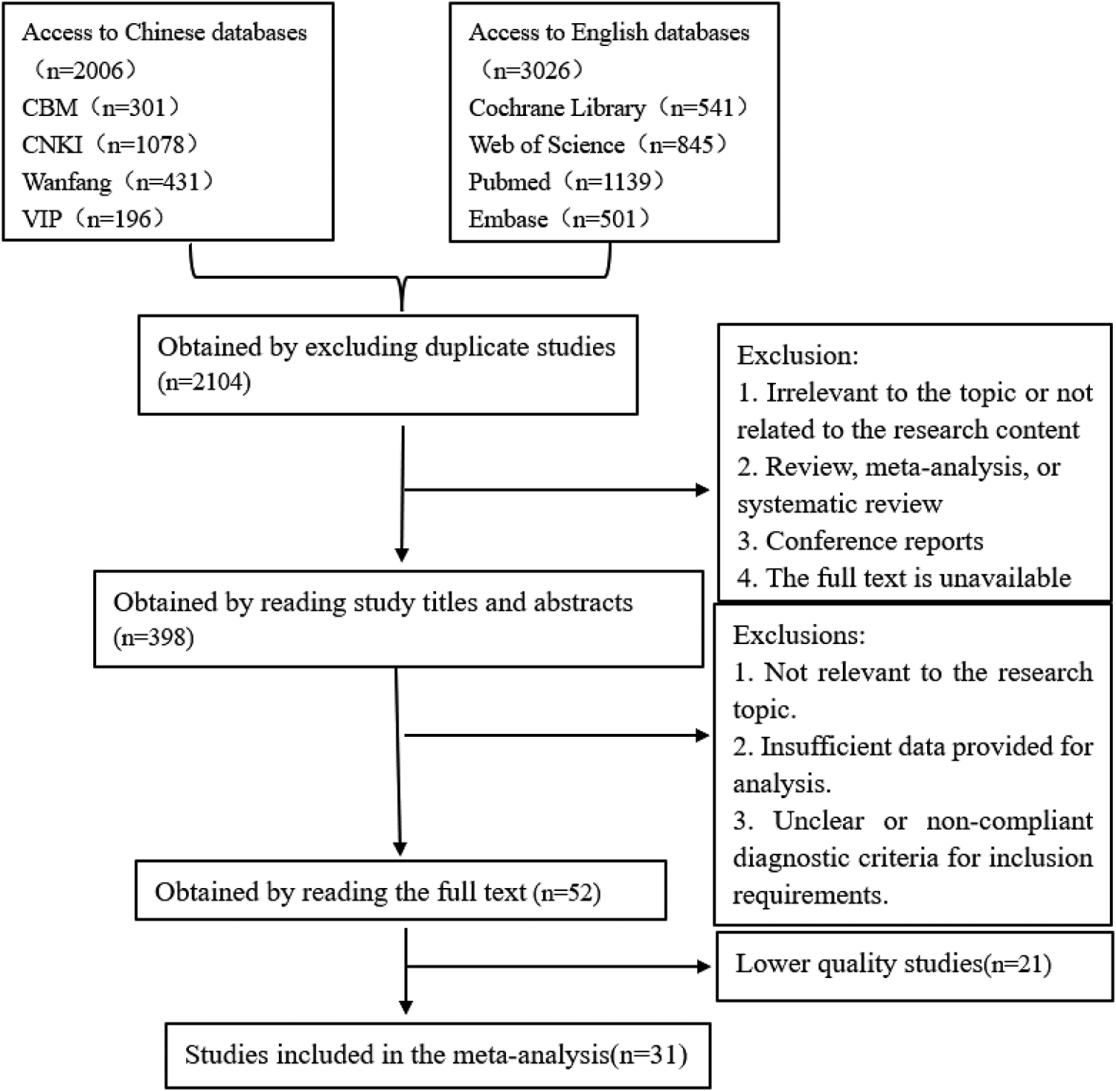
Flowchart of study search and selection.

The systematic review identified 3,032 articles. Following the screening process outlined in Figure 1, 31 studies were ultimately included, of which 9 were prospective cohort studies, and 22 were retrospective cohort studies.

### 3.2 Study quality and characteristics

Quality assessment of the included studies based on the NOS (Newcastle-Ottawa Scale) showed scores ≥7 for all 31 selected articles (Table 1) (16–46). The primary studies was conducted in Asia, the Americas, Europe, Oceania, and Africa. Due to the search results of the Chinese datebases, 14 papers from China and 4 from Africa have the highest participation. Other sources include the United States, Ireland, the United Kingdom, Korea, Pakistan, Somalia, the United Arab Emirates, and Palestine. The publication years of the included studies ranged from 1992 to 2023. A total of 1,204,017 pregnant women were included, with 204,030 cases in the anemia group. The basic characteristics of each study are presented in Table 2.

**Table 1:** Quality assessment of the included studies.

	Representativeness of the exposed cohor	Selection of the nonexposed cohort	Clear diagnostic criteria	Demonstration that outcome of interest was not present at start of study	Control for comparability of major confounders	Control for comparability of minor confounders	Assessment of outcome	ow-up long enough for outcomes to occur	Adequacy of follow up of cohorts	Total points
Adil 2023	1	1	1	1	0	1	1	1	1	8
Amalia 2005	1	1	1	1	0	1	1	1	1	8
Ana 2019	1	1	1	1	1	1	1	1	1	9
Cao 2022	1	1	1	1	0	1	1	1	1	8
Chen 2023	1	1	1	1	1	1	1	1	1	9
Chu 2019	1	1	1	1	1	0	1	1	1	8
Farah 2004	1	1	1	1	1	1	1	1	1	9
Fareh 2009	1	1	1	1	1	0	1	1	1	8
Gwinyai 2015	1	1	1	1	1	0	1	1	1	8
Henna 2003	1	1	1	1	1	1	1	1	1	9
Jin 2022	1	1	1	1	1	1	1	1	1	9
Kora 2006	1	1	1	1	1	0	1	0	1	7
Lin 2018	1	1	1	1	0	0	1	1	1	7
Lior 2015	1	1	1	1	1	1	1	1	1	9
Lu 2014	1	1	1	1	1	1	1	1	1	9
Ma 2020	1	1	1	1	0.5	1	1	1	1	8.5
Manpreet 2015	1	1	1	1	0	1	1	1	1	8
Melaku 2022	1	1	1	1	0	0	1	1	1	7
Ramya 2017	1	1	1	1	1	1	1	1	1	9
Rukuni 2016	1	1	1	1	0	0	1	1	1	7
Sari 2014	1	1	1	1	1	0	1	1	1	8
Shweta 2019	1	1	1	1	0	0	1	1	1	7
Sun 2021	1	1	1	1	1	0	1	1	1	8
Tai 2018	1	1	1	1	0	1	1	1	1	8
Theresa 1992	1	1	1	1	0	0	1	1	1	7
Tian 2023	1	1	1	1	0	1	1	1	1	8
Yi 2013	1	1	1	1	0	0	1	1	1	7
Yu 2017	1	1	1	1	0	0	1	1	1	7
Zhao 2021	1	1	1	1	0	0	1	1	1	7
Zhao 2022	1	1	1	1	0.5	0	1	1	1	7.5
Zheng 2022	1	1	1	1	0	1	1	1	1	8

**Table 2:** The characteristics of studies included in the meta-analysis.

Author, year	Location	Type of research	Research time	Sample size	Anemia size	Study outcomes involved
Adil 2023	Somali	prospective	2022.5–2022.12	1,186	769	4, 7, 8, 9, 11, 13
Amalia 2005	Palestine	retrospective	1988–2002	1,53,396	13,204	4, 7, 10, 11
Ana 2019	Brazil	prospective	2013.1–2017.3	622	155	1, 11
Cao 2022	China	retrospective	2018.11–2019.11	102	20	3, 7, 8, 9, 10, 11, 13
Chen 2023	China	prospective	2013.3–2020.1	34,087	5,572	1, 2, 7, 11, 12, 13
Chu 2019	China-Taiwan	retrospective	2001–2008	13,026	1,795	2, 4, 5, 7, 8, 10, 11, 12
Farah 2004	Pakistan	prospective	2001.10–2002.10	629	313	7, 11
Fareh 2009	UAE	retrospective	\	200	100	7, 8
Gwinyai 2015	Australia, Ireland, New Zealand, United Kingdom	retrospective	2004.11–2011.2	5,609	125	4, 7, 10, 11, 12
Henna 2003	Finland	retrospective	1990–2000	22,799	597	4, 6, 7, 8, 10, 11, 12
Jin 2022	China	retrospective	2017.1–2021.12	800	400	1, 3, 5, 7, 9, 10, 11
Kora 2006	India	prospective	2004.3–2006.2	200	100	4, 5, 6, 7, 8, 10, 11
Lin 2018	China	retrospective	2013.6–2015.5	39,454	6,485	1, 2, 4, 5, 6, 7, 9, 11
Lior 2015	Palestine	retrospective	2005.7–2012.12	75,660	7,977	4, 7, 8, 9, 10, 11, 12
Lu 2014	China	retrospective	2011.1–2014.1	585	285	4, 5, 8
Ma 2020	China	retrospective	2018.2–2019.12	122	61	7, 8, 10, 11
Manpreet 2015	India	prospective	2012.1–2012.6	100	50	4, 7, 11
Melaku 2022	Ethiopia	retrospective	2019.2–2019.4	211	34	11
Ramya 2017	India	prospective	2014.11–2016.9	400	200	4, 7, 8, 9, 11
Rukuni 2016	Scotland	retrospective	1995–2012	80,422	7,475	1, 3, 6, 7, 8, 11
Sari 2014	Finland	retrospective	2006–2010	2,89,967	6,830	4, 7, 12, 13
Shweta 2019	India	retrospective	2016.9–2017.12	515	404	7, 9, 11
Sun 2021	China	retrospective	2016.1–2019.7	46,578	14,078	7, 10, 11, 12
Tai 2018	China	retrospective	2015.1–2016.4	450	225	1, 2, 4, 5, 7, 8, 9, 10, 11
Theresa 1992	America	prospective	\	779	217	7, 11, 12
Tian 2023	China-Taiwan	retrospective	2014.1–2021.12	3,62,023	1,26,636	2, 3, 4, 6, 7, 8, 12
Yi 2013	Korea	retrospective	1997–1999	70,895	8,581	7, 11, 12
Yu 2017	China	retrospective	2014.6–2016.11	376	115	1, 4, 7, 8, 9, 10, 11
Zhao 2021	China	prospective	2018.1–2019.6	720	240	1, 4, 7, 8, 9, 10
Zhao 2022	China	retrospective	2016.1–2021.10	721	104	1, 4, 7, 8, 9, 10
Zheng 2022	China	retrospective	2018.10–2021.10	1,383	883	1, 2, 3, 5, 7, 8, 9, 10

(1), Gestational hypertension; (2), Gestational diabetes mellitus(GDM); (3), Puerperal infection; (4), Cesarean section; (5), Premature rupture of membranes(PROM); (6), Pre-eclampsia; (7), Preterm birth; (8), Postpartum hemorrhage; (9), Fetal distress; (10), Neonatal asphyxia; (11), Low birth weight infant(LBW); (12), Small for gestational age (SGA); (13), Congenital malformation.

### 3.3 Meta-analysis results

#### 3.3.1 Meta-analysis of the relationship between anemia and adverse pregnancy outcomes

The pooled meta-analysis results for all possible adverse pregnancy outcomes are presented in Table 3. A total of 10 studies (18, 20, 26, 28, 35, 39, 43–46) investigated the relationship between anemia during pregnancy and pregnancy hypertension. The analysis showed that anemia during pregnancy may increase the risk of hypertension-induced pregnancy. Significant heterogeneity was found among the studies (*I^2^* = 86%, *P* < 0.0001). The meta-analysis revealed that the rate of pregnancy-induced hypertension was higher in the anemia group compared to the control group, with a pooled *RR* of 1.33 (95% *CI* 1.02, 1.74). The forest and funnel plots are shown in Supplementary Figures S1 and S2.

**Table 3:** Meta-analysis of the association between anemia during pregnancy and the risk of adverse maternal outcomes.

Outcome	No. of studies	Summary *RR* (95% *CI*)	Test for heterogeneity	
			*I^2^*	*P*-value
Gestational hypertension	10	1.33 (1.02, 1.74)	86%	<0.0001
GDM	5	0.81 (0.77, 0.84)	0	0.74
Puerperal infection	5	1.72 (0.91, 3.26)	79%	0.0009
Cesarean section	17	1.28 (1.14, 1.44)	98%	<0.0001
PROM	7	1.94 (1.26, 3.00)	92%	<0.0001
Pre-eclampsia	5	1.03 (0.87, 1.21)	76%	0.002
Premature delivery	28	1.51 (1.33, 1.72)	93%	<0.0001
Postpartum hemorrhage	17	2.76 (1.63, 4.66)	97%	<0.0001
Fetal distress	12	1.13 (0.94, 1.36)	83%	<0.0001
Neonatal asphyxia	14	1.21 (1.07, 1.37)	44%	0.04
LBW	24	1.40 (1.19, 1.63)	94%	<0.001
SGA	10	0.96 (0.80, 1.15)	98%	<0.001

CI, confidence interval; RR, relative risk; *I^2^*, heterogeneity; GDM, gestational diabetes mellitus; PROM, premature rupture of membranes; LBW, low birth weight; SGA, small for gestational age.

Seventeen studies (16, 17, 21, 24, 25, 27–30, 32, 34, 36, 39, 42–45) examined the association between anemia during pregnancy and mode of delivery. Anemia during pregnancy may increase the probability of cesarean section. Significant heterogeneity was observed among the studies (*I^2^* = 98%, *P* < 0.0001), indicating that pregnant women with anemia had a higher likelihood of undergoing cesarean section compared to the control group, with a pooled *RR* of 1.28 (95% *CI* 1.14, 1.44). The forest and funnel plots are shown in Supplementary Figures S3 and S4.

Seven studies (21, 26–28, 30, 39, 46) investigated the relationship between anemia during pregnancy and PROM. Anemia during pregnancy will increase the risk of PROM. Significant heterogeneity was present among the studies (*I^2^* = 92%, *P* < 0.0001), and pregnant women with anemia had a significantly higher probability of experiencing PROM compared to the control group, with a pooled *RR* of 1.94 (95% *CI* 1.26, 3.00). The forest and funnel plots are shown in Supplementary Figures S5 and S6.

Twenty-eight studies (16, 17, 19–29, 31, 32, 34–46) explored the association between pregnancy with anemia and preterm birth. Anemia during pregnancy will increase the risk of preterm birth. Significant heterogeneity was detected among the studies (*I^2^* = 95%, *P* < 0.0001), indicating that pregnant women with anemia had a significantly higher risk of preterm birth compared to the control group, with a pooled *RR* of 1.51 (95% *CI* 1.33, 1.72). The forest and funnel plots are shown in Supplementary Figures S7 and S8.

Seventeen studies (16, 19, 21, 23, 25, 27, 29–31, 34, 35, 39, 43–46) investigated the relationship between pregnancy with anemia and postpartum hemorrhage. Anemia during pregnancy will increase the risk of postpartum hemorrhage.Significant heterogeneity was found among the studies (*I^2^* = 97%, *P* < 0.0001), and pregnant women with anemia had a significantly higher probability of experiencing postpartum hemorrhage compared to the control group, with a pooled *RR* of 2.76 (95% *CI* 1.63, 4.66). The forest and funnel plots are shown in Supplementary Figures S9 and S10.

Fifteen studies (17, 19, 21, 22, 24–27, 29, 31, 38, 43–46) examined the association between pregnancy with anemia and neonatal asphyxia. Anemia during pregnancy may increase the risk of neonatal asphyxia. Sensitivity analysis revealed that there was high heterogeneity before removing the study by Lior et al. (29) (*I^2^* *=* 74%). Therefore, it was excluded from the analysis. There was lower heterogeneity among the studies (*I^2^* = 44%, *P* = 0.04), and the incidence of neonatal asphyxia was higher in the anemia group compared to the control group, with a pooled *RR* of 1.21 (95% *CI* 1.07, 1.37). The forest and funnel plots are shown in Supplementary Figures S11 and S12.

Twenty-four studies (16–22, 24–29, 31–35, 37–40, 42, 43) investigated the relationship between pregnancy with anemia and LBW. Anemia during pregnancy may increase the risk of LBW. Significant heterogeneity was observed among the studies (*I^2^* = 94%, *P* < 0.0001), and the incidence of LBW was significantly higher in the anemia group compared to the control group, with a pooled *RR* of 1.40 (95% *CI* 1.19, 1.63). The forest and funnel plots are shown in Supplementary Figures S13 and S14.

Through the method of sequentially excluding individual studies for sensitivity analysis, the results indicated that the study by Tian et al. (31) was the source of heterogeneity for GDM (*I^2^* = 97%, *P* < 0.001). After excluding the study by Tian et al, the heterogeneity became 0, but the result showed a meaningless negative correlation with *RR* (95% *CI*) of 0.81 (0.77, 0.84).

The meta-analysis indicated that there was no statistically significant difference in the risk of puerperal infection, pre-eclampsia, fetal distress and SGA between the anemia group and the non-anemia group. The pooled *RR*s (95% *CI*s) were 1.72 (0.91, 3.26), 1.03 (0.87, 1.21), 2.76 (1.63, 4.66) and 0.96 (0.80, 1.15) respectively. The forest and funnel plots are shown in Supplementary Figures S15–S22.

#### 3.3.2 Subgroup analysis

As shown in Table 4, meta-regression analyses were conducted on subgroups of pregnancy outcomes with high heterogeneity, including postpartum hemorrhage, PROM, preterm birth, LBW, cesarean section and gestational hypertension based on national income levels, study types and sample sizes. The results indicated that income levels may be a source of heterogeneity for postpartum hemorrhage (*P* = 0.02), premature delivery (*P* < 0.001) and cesarean section (*P* < 0.001). Study design types may be a source of heterogeneity for postpartum hemorrhage (*P* < 0.001) and preterm birth (*P* < 0.001). Sample sizes may be a source of heterogeneity for preterm birth (*P* < 0.001) and low birth weight (*P* < 0.001).

**Table 4:** Subgroup analysis results.

	No. of studies	*RR* (95% *CI*)	Test for heterogeneity		Meta-regression (*P*-value)
			*I^2^*	*P-*value	
Postpartum hemorrhage					
Country income category					
High-income	6	3.81 [3.60, 4.03]	99%	<0.001	0.02
Low or middle-income	11	2.84 [2.25, 3.58]	79%	<0.001	
Study design					
Prospective	4	2.06 [1.47, 2.88]	81%	0.001	<0.001
Retrospective	13	3.71 [3.51, 3.93]	98%	<0.001	
Sample size					
<800	10	4.11 [3.07, 5.50]	70%	<0.001	0.52
≥800	7	3.72 [3.52, 3.94]	99%	<0.001	
PROM					
Country income category					
High-income	1	1.56 [1.12, 2.16]	/	/	0.37
Low or middle-income	6	2.06 [1.23, 3.45]	93%	<0.001	
Study design					
Prospective	1	2.25 [0.72, 7.07]	/	/	0.80
Retrospective	6	1.92 [1.22, 3.02]	93%	<0.001	
Sample size					
<800	3	2.96 [0.65, 13.58]	91%	<0.001	0.40
≥800	4	1.51 [1.01, 2.26]	91%	<0.001	
Premature delivery					
Country income category					
High-income	10	1.19 [1.15, 1.24]	76%	<0.001	<0.001
Low or middle-income	18	1.03 [1.01, 1.06]	97%	<0.001	
Study design					
Prospective	8	1.74 [1.62, 1.87]	87%	0.001	<0.001
Retrospective	20	1.03 [1.01, 1.06]	95%	<0.001	
Sample size					
<800	13	2.78 [2.34, 3.31]	77%	<0.001	<0.001
≥800	15	1.04 [1.02, 1.07]	96%	<0.001	
LBW					
Country income category					
High-income	8	1.07 [1.03, 1.12]	81%	<0.001	<0.001
Low or middle-income	16	1.53 [1.44, 1.61]	93%	<0.001	
Study design					
Prospective	8	2.08 [1.93, 2.24]	64%	0.007	<0.001
Retrospective	16	1.08 [1.04, 1.12]	88%	<0.001	
Sample size					
<800	12	1.84 [1.55, 2.18]	48%	0.03	<0.001
≥800	12	1.20 [1.16, 1.24]	97%	<0.001	
Cesarean section					
Country income category					
High-income	7	1.26 [1.04, 1.53]	99%	<0.001	<0.001
Low or middle-income	10	1.35 [1.07, 1.70]	91%	<0.001	
Study design					
Prospective	8	1.74 [1.42, 2.13]	80%	<0.001	0.07
Retrospective	9	1.44 [1.42, 1.46]	98%	<0.001	
Sample size					
<800	5	1.58 [1.34, 1.85]	92%	<0.001	0.27
≥800	12	1.44 [1.42, 1.46]	99%	<0.001	
Gestational hypertension					
Country income category					
High-income	1	0.77 [0.71, 0.84]	/	/	0.001
Low or middle-income	9	1.47 [1.11, 1.94]	72%	<0.001	
Study design					
Prospective	3	1.62 [0.73, 3.62]	78%	<0.001	0.53
Retrospective	7	1.23 [0.91, 1.67]	82%	<0.001	
Sample size					
<800	5	2.13 [1.08, 4.17]	60%	0.04	0.07
≥800	5	1.10 [0.83, 1.44]	90%	<0.001	

CI, confidence interval; RR, relative risk; *I^2^*, heterogeneity; PROM, premature rupture of membranes; LBW, low birth weight;.

*P*-value, All heterogeneity values were significant at *P* < 0.05 level.

## 4 Discussion

Anemia is one of the common complications during pregnancy, even among pregnant women in developed countries (47). Due to the increased demand for nutrients during pregnancy, some pregnant women may experience anemia due to inadequate daily intake of nutrients or lower nutrient utilization rates (48). Chowdhury et al's (49) study indicated maternal age, BMI levels, economic status and educational level are associated with hemoglobin levels during pregnancy. Previous research has suggested that severe anemia during pregnancy, resulting in decreased hemoglobin levels and reduced oxygen-carrying capacity of the blood, can lead to various adverse pregnancy outcomes, including pregnancy-induced hypertension, miscarriage, preterm birth and LBW (50, 51).

This study found that pregnant women with anemia were at higher risk of developing pregnancy-induced hypertension, cesarean section, PROM, preterm birth, postpartum hemorrhage, neonatal asphyxia and LBW compared to non-anemic pregnant women with respective *RR* values of 1.33, 1.28, 1.94, 1.51, 2.76, 1.21 and 1.40. In terms of risk severity, the order from highest to lowest was postpartum hemorrhage, PROM, preterm birth, LBW, cesarean section, pregnancy-induced hypertension and neonatal asphyxia.

Our study corroborates previous research in identifying several adverse pregnancy outcomes associated with anemia. Xiong et al's (10) meta-analysis on the relationship between anemia during pregnancy and pregnancy outcomes investigated the *OR*s (95% *CI*s) of early pregnancy anemia with preterm birth, LBW, fetal growth restriction and hypertension, which were 1.32 (1.01, 1.74), 1.39 (0.70, 2.74), 1.01 (0.73, 1.38) and 0.80 (0.53, 1.20) respectively. In comparison, this study identified four adverse outcomes related to pregnancy anemia: PROM, cesarean section, postpartum hemorrhage and neonatal asphyxia. While Xiong et al' 's (10) study did not find a statistically significant association between anemia and pregnancy-induced hypertension, this study demonstrated a strong correlation between anemia during pregnancy and pregnancy-induced hypertension, with an *RR* value of 1.89. This difference may be attributed to the inclusion of only two relevant studies with potentially lower credibility in Xiong et al's analysis (52, 53). Our study encompassed a more comprehensive set of relevant studies, enhancing the reliability of our results. Additionally, some studies treated hypertension as a confounding factor and excluded it from their analysis, potentially influencing the results. When pregnant women have anemia, the decreased oxygen-carrying capacity of the blood leads to increased cardiovascular and peripheral vascular pressure to meet the demands for blood supply, thereby increasing the risk of hypertension (54).

The mechanisms underlying the adverse pregnancy outcomes in anemic pregnant women are multifactorial and biologically plausible. The decreased oxygen-carrying capacity of the blood due to anemia leads to compensatory physiological responses (55, 56). For instance, the increased cardiovascular and peripheral vascular pressure to meet the oxygen demands of the fetus can precipitate hypertension (57, 58). Postpartum hemorrhage is a significant cause of maternal mortality. Omotayo et al's (59) study showed that severe anemia during pregnancy increases the risk of postpartum hemorrhage, and there is no significant correlation between mild to moderate anemia and postpartum hemorrhage. The mechanism behind this may be related to iron deficiency anemia and increased secretion of nitric oxide. During pregnancy, trophoblasts and placental cells secrete nitric oxide through paracrine action, which then binds with nitric oxide synthase in the uterine muscle layer. This process plays a crucial role in maintaining the stability of the uterus. In the case of anemia, this process is intensified, leading to a decrease in the contraction function of uterine smooth muscles, thereby causing uterine muscle weakness and increasing the risk of postpartum uterine bleeding (60). Nur et al (61). also confirmed in a case-control study that anemia is a significant risk factor for postpartum hemorrhage.

Fetal hypoxia, a consequence of maternal anemia, is a key contributor to neonatal asphyxia. Maternal anemia causes a reduction in hemoglobin levels in the maternal blood, decreasing the blood's capacity to carry oxygen and affecting the fetus's ability to obtain sufficient oxygen in the uterus. When the fetus experiences hypoxia in the maternal uterus, it may result in asphyxia (62, 63). Additionally, this study demonstrates a strong association between maternal anemia during pregnancy and LBW and preterm birth with *RR* values of 1.40 and 1.51, respectively. Allen's (64) research suggests that placental insufficiency in anemic patients leading to hypoxia is considered one of the mechanisms causing LBW. Maternal anemia is often accompanied by inadequate nutrient intake and insufficient placental blood supply. Due to the lack of essential nutrients, the metabolic capacity of pregnant women's cells decreases, affecting fetal development and growth, leading to LBW (65). Furthermore, when the immune system function of newborns is immature, there is an increased risk of complications such as respiratory infections, indirectly raising the likelihood of neonatal asphyxia (66).

In general, maternal anemia during pregnancy impacts fetal oxygen supply and overall health, potentially leading to inadequate uterine contractions that can affect fetal growth and development. This condition may trigger preterm birth or delivery complications such as placental abruption, PROM, among others (67–69). Apart from oxidative damage, another known biological mechanism indicates that anemia and iron deficiency stimulate the synthesis of corticotropin-releasing hormone (CRH), leading to stress responses in both the mother and the fetus (70). Elevated levels of CRH are major risk factors for preterm birth, gestational hypertension, preeclampsia and PROM (64, 71). This study did not find an association between maternal anemia during pregnancy and gestational diabetes, puerperal infections, fetal distress, etc., which may be related to unpublished negative study results or differences in literature diagnostic criteria. Further, future high-quality research and relevant data are needed for supplementation and improvement.

The high heterogeneity observed in our meta-analysis, as indicated by the *I*^2^ values, warrants further exploration. Subgroup analyses based on factors such as country income levels, study design, and sample size have provided valuable insights. For example, income levels influenced the risk of postpartum hemorrhage, preterm birth, and cesarean section. In high-income countries, the risk of postpartum hemorrhage was higher, potentially due to differences in healthcare utilization and management practices. Study design also emerged as a significant source of heterogeneity, with retrospective studies often showing different results compared to prospective ones. This could be attributed to differences in data collection methods, recall bias in retrospective studies, and the ability to control for confounding factors. Sample size differences further contributed to heterogeneity, particularly in outcomes like preterm birth and LBW. Smaller studies may be more susceptible to sampling variability and less likely to capture the full spectrum of associations.

The strength of this study lies in focusing solely on the pregnancy outcomes of singleton anemic women. This is because twin or multiple pregnancies carry a higher risk of adverse perinatal outcomes. If there were a significant number of multiple pregnancies in the anemic group, it would lead to selection bias and magnify the impact of anemia on adverse pregnancy outcomes. Our study also has several limitations that should be considered when interpreting the results. On the one hand, the reliance on publicly available Chinese and English literature may have introduced selection bias, potentially overlooking relevant studies in other languages or unpublished research. The exclusion of specific types of anemia, such as aplastic anemia and thalassemia, limits the generalizability of our findings. On the other hand, while we standardized certain factors like singleton pregnancies, the lack of control for other confounding variables such as parity and BMI may have affected the accuracy of our estimates. Future studies should expand the literature search to include non-English and non-Chinese databases and grey literature sources more comprehensively. Collaborations with international research groups could also help in accessing a broader range of studies. In addition, future researchs can specifically investigate the impact of different types of anemia on pregnancy outcomes. This could involve conducting separate meta-analyses for each major type of anemia or including detailed subgroup analyses within a comprehensive study.

## 5 Conclusion

Maternal anemia is associated with an increased risk of adverse pregnancy outcomes. Anemia during pregnancy poses significant risks, not only leading to poor health conditions for pregnant women, premature rupture of membranes and postpartum hemorrhage but also being detrimental to fetal development, resulting in outcomes such as low birth weight, preterm birth and neonatal asphyxia. For pregnant women, special attention should be paid to supplementing iron. Adopting excellent nutritional management measures plays an essential supportive role in preventing the occurrence of pregnancy-induced anemia and avoiding the worsening of anemia.
