# Supplementary material for: Anemia during pregnancy and adverse pregnancy outcomes: a systematic review and meta-analysis of cohort studies
Source: Front Glob Womens Health. 2025 Jan 31;6:1502585. doi: 10.3389/fgwh.2025.1502585 (PMC11825799; doi:10.3389/fgwh.2025.1502585)
Supplement: Supplementary file 1 [file Supplementaryfile1.docx]

Forest map of the effect of gestational anemia on Supplementary Material

## Supplementary Figure
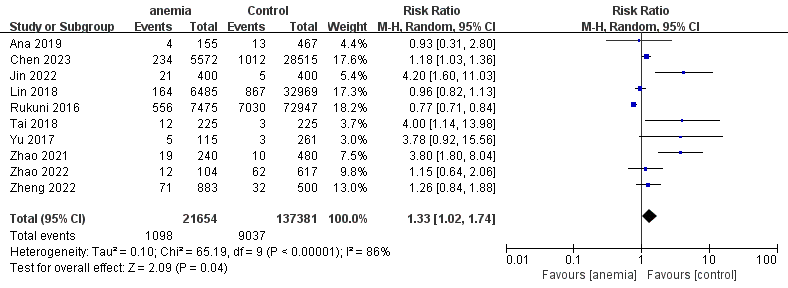
Supplementary Figure 1 Forest map of the effect of anemia during pregnancy on gestational hypertension
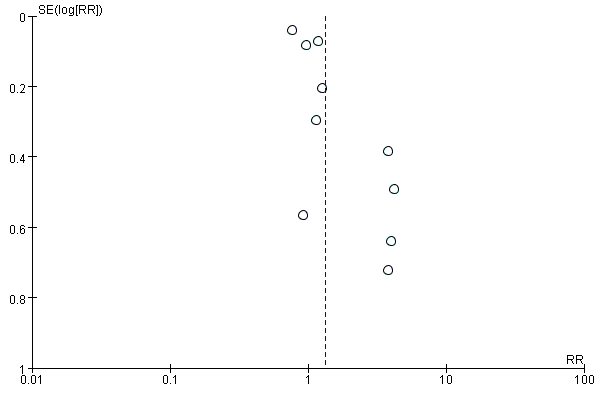
Supplementary Figure 2 Funnel plot of the effect of anemia during pregnancy on gestational hypertension


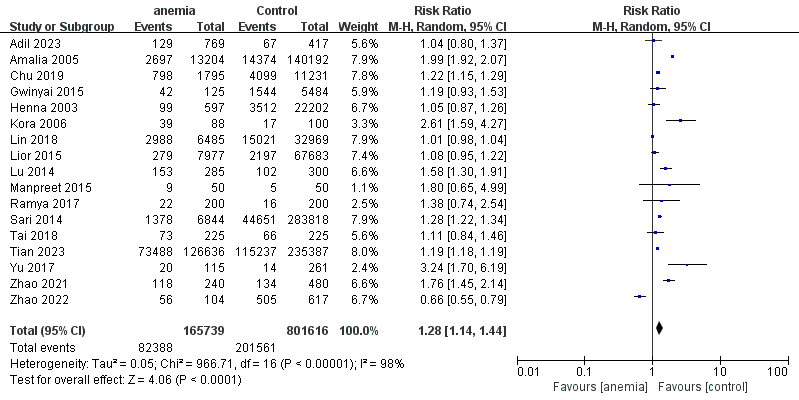


Supplementary Figure 3 Forest map of the effect of anemia during pregnancy on caesarean section


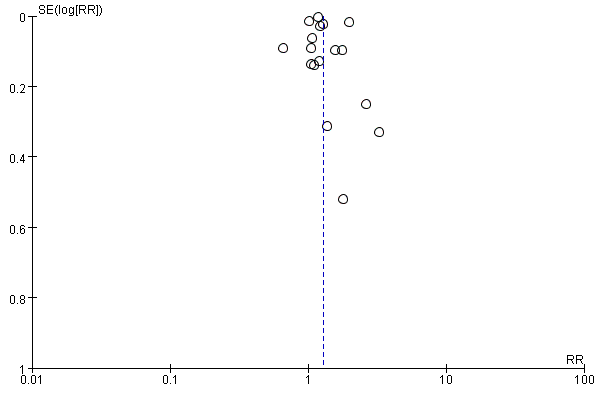


Supplementary Figure 4 Funnel plot of the effect of anemia during pregnancy on caesarean section

##
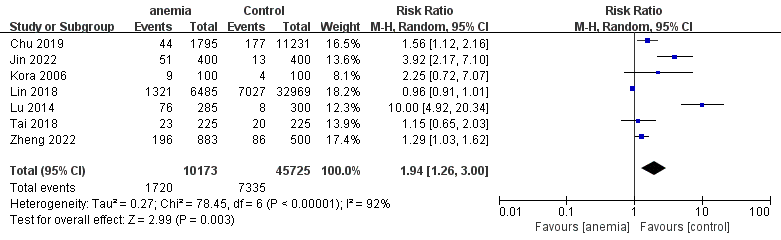


Supplementary Figure 5 Forest map of the effect of anemia during pregnancy on PROM


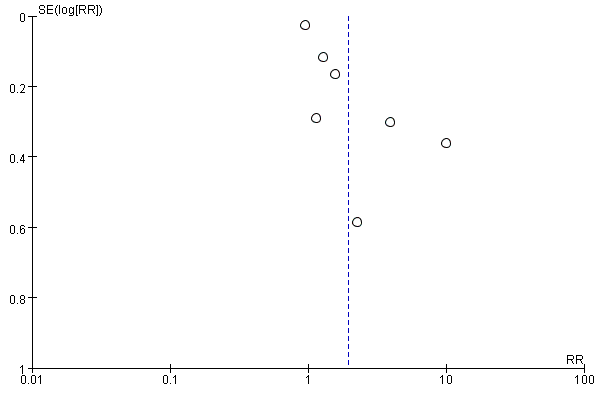


Supplementary Figure 6 Funnel plot of the effect of anemia during pregnancy on PROM


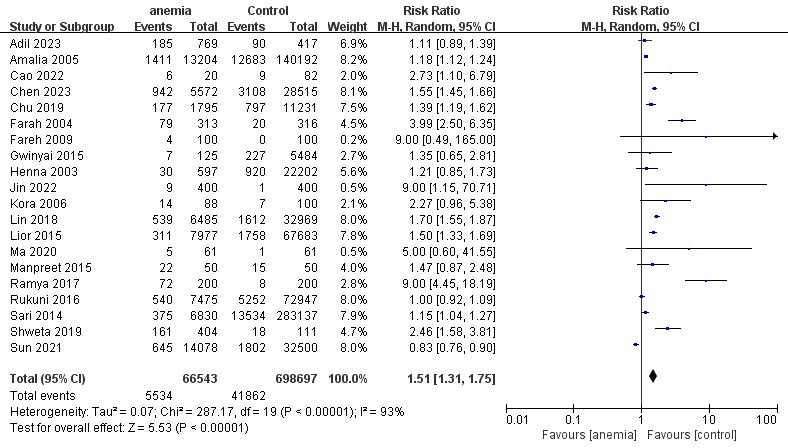


Supplementary Figure 7 Forest map of the effect of anemia during pregnancy on premature delivery


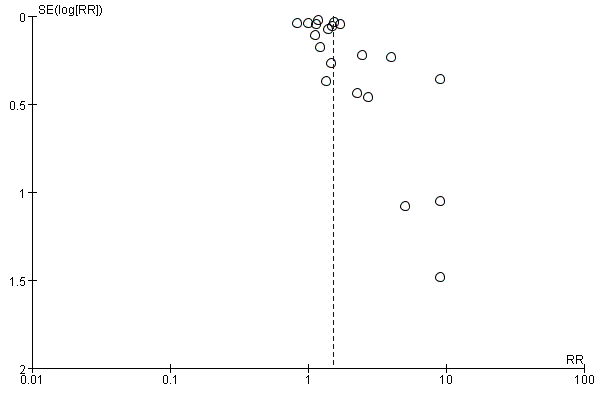


Supplementary Figure 8 Funnel plot of the effect of anemia during pregnancy on premature delivery

##
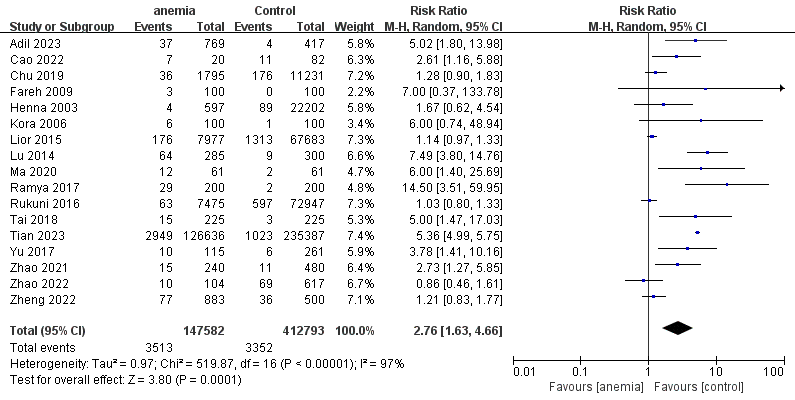


Supplementary Figure 9 Forest map of the effect of anemia during pregnancy on postpartum hemorrhage


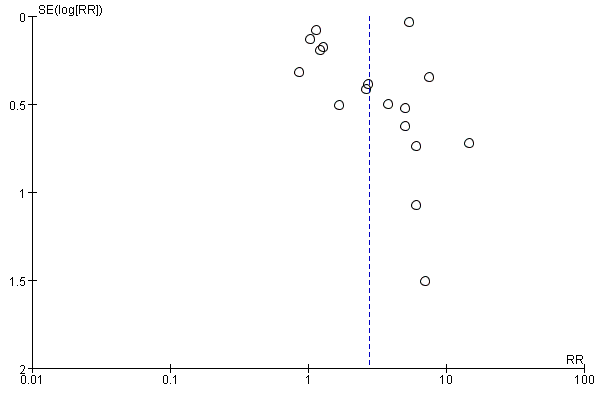


Supplementary Figure 10 Funnel plot of the effect of anemia during pregnancy on postpartum hemorrhage

##
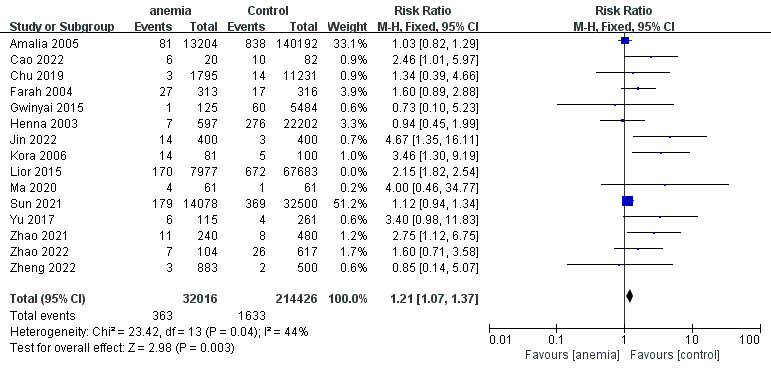


Supplementary Figure 11 Forest map of the effect of anemia during pregnancy on neonatal asphyxia
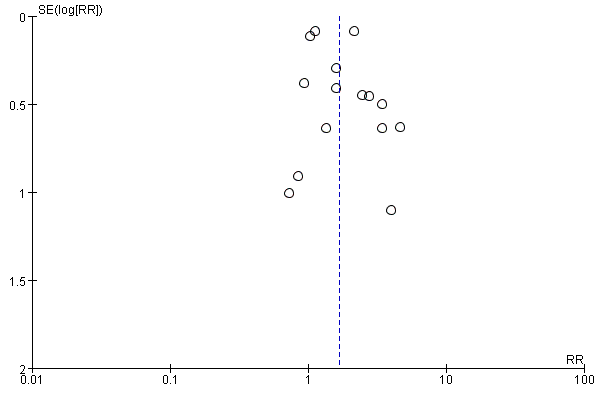


Supplementary Figure 12 Funnel plot of the effect of anemia during pregnancy on neonatal asphyxia


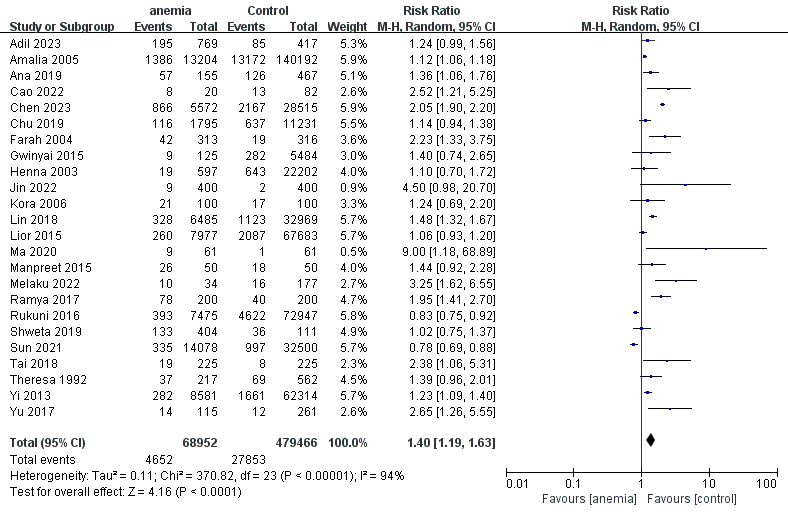


Supplementary Figure 13 Forest map of the effect of anemia during pregnancy on LBW


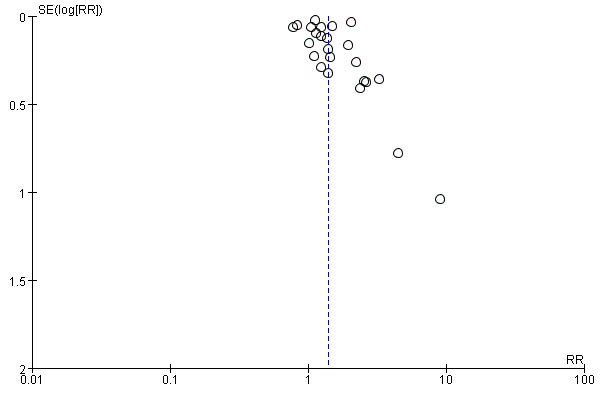


Supplementary Figure 14 Funnel plot of the effect of anemia during pregnancy on LBW


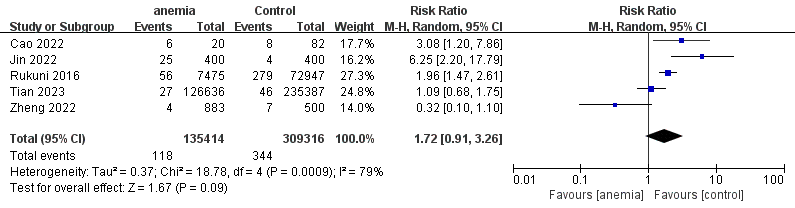


Supplementary Figure 15 Forest map of the effect of anemia during pregnancy on puerperal infection


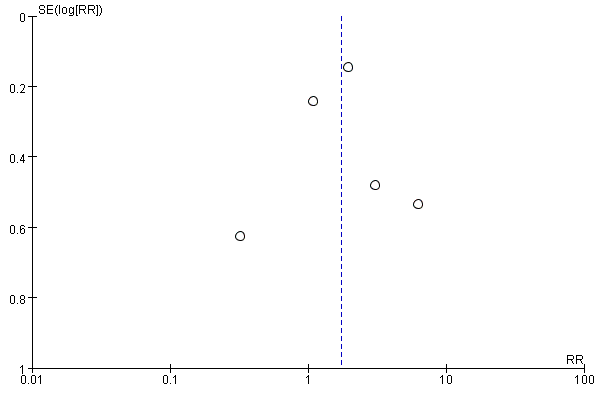


Supplementary Figure 16 Funnel plot of the effect of anemia during pregnancy on puerperal infection


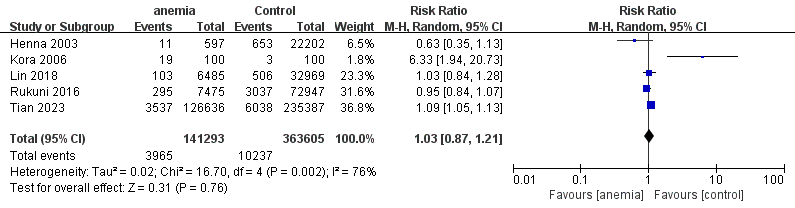


Supplementary Figure 17 Forest map of the effect of anemia during pregnancy on pre-eclampsia


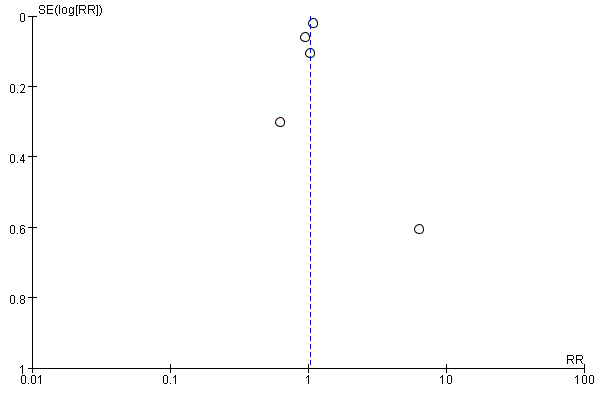


Supplementary Figure 18 Funnel plot of the effect of anemia during pregnancy on pre-eclampsia


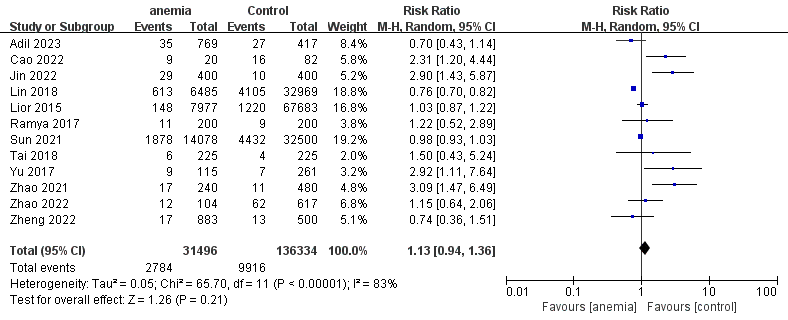


Supplementary Figure 19 Forest map of the effect of anemia during pregnancy on fetal distress


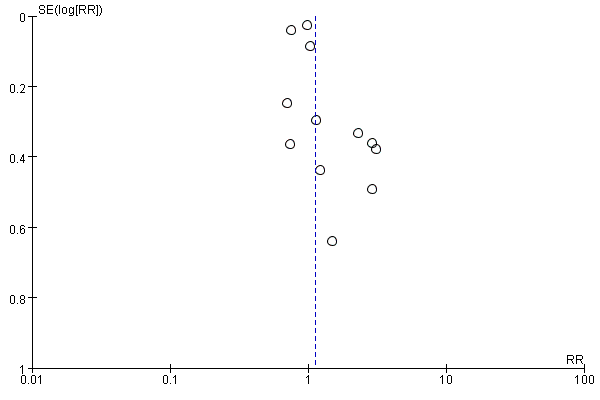


Supplementary Figure 20 Funnel plot of the effect of anemia during pregnancy on fetal distress


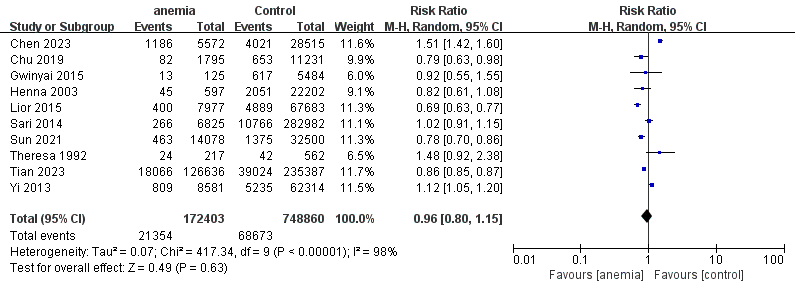


Supplementary Figure 21 Forest map of the effect of anemia during pregnancy on SGA


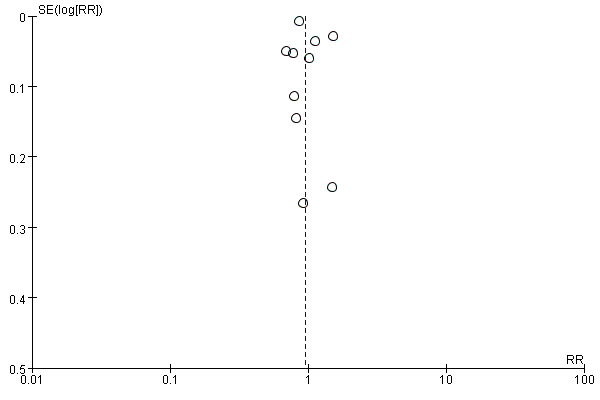


Supplementary Figure 22 Funnel plot of the effect of anemia during pregnancy on SGA
